# Supplementary material for: Is addressing violence against women prioritised in health policies? Findings from a WHO policies database
Source: PLOS Glob Public Health. 2024 Feb 16;4(2):e0002504. doi: 10.1371/journal.pgph.0002504 (PMC10871498; doi:10.1371/journal.pgph.0002504)
Supplement: S1 Table — (DOCX) [file pgph.0002504.s001.docx]

| **Identification of intimate partner violence (IPV)** | WHO recommends that countries include a clinical inquiry approach to identifying IPV, but that they should not do universal screening^^[[1]](#footnote-1)^^. |
| --- | --- |
| **First line support** | WHO recommends offering every woman who discloses IPV or sexual violence first line support – a woman-centred adaptation of psychological first aid, which involves responding to the practical and emotional needs of the survivor and can be delivered using a job aid called LIVES^^[[2]](#footnote-2)^^. |
| **Post-rape care services** | WHO recommends that survivors of sexual assault be offered a package of post-rape care services including: emergency contraception (EC) within 120 hours of the assault; HIV post exposure prophylaxis (PEP) within 72 hours of the assault; STI treatment or prophylaxis; safe abortion if the woman is pregnant and to the full extent of the law. |
| **Mental health care** | For both IPV and sexual assault, WHO recommends offering an assessment of mental health, treatment and/or referral to mental health services by a specialist for those suffering from mental health conditions such as depression or post-traumatic stress disorder (PTSD). |

S1 Table: WHO-recommended services for survivors of VAW (WHO, 2013)

1. Clinical enquiry includes the use of questions based on the presenting conditions, history and, where appropriate, examination. Universal screening is a large-scale assessment of whole population groups to identify individuals that have experienced violence. [↑](#footnote-ref-1)
2. LIVES is an acronym for: listen with empathy, inquire about her needs, validate her experience, enhance her safety, and facilitate social support (WHO, 2014) [↑](#footnote-ref-2)
