# Supplementary material for: Is addressing violence against women prioritised in health policies? Findings from a WHO policies database
Source: PLOS Glob Public Health. 2024 Feb 16;4(2):e0002504. doi: 10.1371/journal.pgph.0002504 (PMC10871498; doi:10.1371/journal.pgph.0002504)
Supplement: S2 Table — (DOCX) [file pgph.0002504.s002.docx]

S2 Table: Proportion of countries with a national health policy that includes VAW response and/or prevention as a strategic priority, a multisectoral VAW policy (including whether this includes the health sector), or clinical guidelines, by SDG regions and sub-regions

| **SDG Region and sub-regions** | **National health policy with VAW response and/or prevention as a strategic focus (%)** | **Multisectoral VAW policy (%)** | **Multisectoral VAW policy that includes health sector (%)** | **Clinical guidelines responding to VAW (%)** |
| --- | --- | --- | --- | --- |
| **Africa SDG region (n=54)** | **39** | **81** | **69** | **44** |
| Northern Africa (n=6) | 0 | 83 | 83 | 67 |
| Sub-Saharan Africa (n=48) | 44 | 81 | 67 | 42 |
| **Americas SDG region (n=35)** | **57** | **80** | **80** | **60** |
| Latin America & the Caribbean (n=33) | 58 | 82 | 82 | 64 |
| North America (n=2) | 50 | 50 | 50 | 0 |
| **Asia SDG sub-region (n=47)** | **17** | **68** | **55** | **47** |
| Central Asia (n=5) | 0 | 20 | 20 | 20 |
| Eastern Asia (n=5) | 0 | 80 | 60 | 0 |
| South-Eastern Asia (n=11) | 18 | 73 | 64 | 55 |
| Southern Asia (n=9) | 56 | 67 | 44 | 78 |
| Western Asia (n=17) | 6 | 76 | 65 | 47 |
| **Europe SDG region (n=42)** | **17** | **95** | **81** | **48** |
| Eastern Europe (n=10) | 20 | 100 | 100 | 20 |
| Northern Europe (n=10) | 10 | 100 | 90 | 50 |
| Southern Europe (n=14) | 21 | 93 | 57 | 64 |
| Western Europe (n=8) | 13 | 88 | 88 | 50 |
| **Oceania SDG region (n=16)** | **63** | **81** | **69** | **38** |
| Australia and New Zealand (n=2) | 100 | 100 | 100 | 100 |
| Melanesia (n=4) | 75 | 100 | 75 | 75 |
| Micronesia (n=5) | 40 | 60 | 40 | 20 |
| Polynesia (n=5) | 60 | 80 | 80 | 0 |
| **Grand Total (n=194)** | **34** | **81** | **70** | **48** |

*n=192 for WB income groups due to some countries not classified into income groups by the WB.

Global average % is the same for SDG region and WB income group region
