# Supplementary material for: Is addressing violence against women prioritised in health policies? Findings from a WHO policies database
Source: PLOS Glob Public Health. 2024 Feb 16;4(2):e0002504. doi: 10.1371/journal.pgph.0002504 (PMC10871498; doi:10.1371/journal.pgph.0002504)
Supplement: S3 Table — (DOCX) [file pgph.0002504.s003.docx]

S3 Table: Proportion of countries with a national health policy that includes VAW as a strategic priority, a multisectoral VAW policy (including whether this includes the health sector), or clinical guidelines, by World Bank income groups

| **World Bank income group** | **National health policy with VAW as strategic focus (%)** | **Multisectoral VAW policy (%)** | **Multisectoral VAW policy that includes health sector (%)** | **Clinical guidelines responding to VAW (%)** |
| --- | --- | --- | --- | --- |
| Low-income (n=29) | 38 | 76 | 66% | 45 |
| Lower-middle income (n=49) | 47 | 82 | 63 | 55 |
| Upper-middle income (n=54) | 37 | 87 | 78 | 50 |
| High-income (n=60) | 18 | 78 | 72 | 43 |
| **Global (192)** | **34** | **81** | **70** | **48** |
