# Supplementary material for: Is addressing violence against women prioritised in health policies? Findings from a WHO policies database
Source: PLOS Glob Public Health. 2024 Feb 16;4(2):e0002504. doi: 10.1371/journal.pgph.0002504 (PMC10871498; doi:10.1371/journal.pgph.0002504)
Supplement: S4 Table — (DOCX) [file pgph.0002504.s004.docx]

S4 Table: Proportion of countries that include clinical enquiry or universal screening in policy, by SDG regions and World Bank income groups

|  | **Universal screening included (%)** | **Clinical enquiry included (%)** | **Universal screening and**  **clinical enquiry included (%)** |
| --- | --- | --- | --- |
| **SDG region** | | | |
| Africa (n=50) | 6 | 20 | 0 |
| Americas (n=34) | 18 | 24 | 3 |
| Asia (n=36) | 6 | 33 | 3 |
| Europe (n=41) | 12 | 22 | 2 |
| Oceania (n=13) | 8 | 23 | 0 |
| **Global (n=174)** | **10** | **24** | **2** |
| **World bank income group** | | | |
| Low income (n=25) | 8 | 24 | 0 |
| Lower middle income (n=45) | 4 | 31 | 2 |
| Upper middle income (n=49) | 12 | 27 | 4 |
| High income (n=54) | 13 | 17 | 0 |
| **Global (n=173)** | **10** | **24** | **2** |

Notes:

i) Policy documents were found for 174 of the 194 countries so n=174 for SDG regions but n=173 for World Bank income groups because one country for which policy documents were found (Cook Islands) is not assigned to a World Bank income group.

ii) There were two other possible answer options used: 'unclear’ or ‘unknown/translation not available'. These are not included in this table because the data in the table is a combination of two indicators (universal screening and clinical enquiry) so there's not a single 'unclear' or ‘unknown/translation not available' group. The total would not equal 100%.
