# Supplementary material for: Is addressing violence against women prioritised in health policies? Findings from a WHO policies database
Source: PLOS Glob Public Health. 2024 Feb 16;4(2):e0002504. doi: 10.1371/journal.pgph.0002504 (PMC10871498; doi:10.1371/journal.pgph.0002504)
Supplement: S6 Table — (DOCX) [file pgph.0002504.s006.docx]

S6 Table: Proportion of countries that include abortion for survivors of VAW in policy, by SDG regions and WB income groups

|  | **Yes, included**  **(%)** | **No, not included**  **(%)** | **Not specified**  **(%)** | **Unclear**  **(%)** | **Unknown - translation not available/ usable (%)** | **Total**  **(%)** |
| --- | --- | --- | --- | --- | --- | --- |
| **SDG region** | | | | | | |
| Africa (n=50) | 16 | 4 | 70 | 8 | 2 | 100 |
| Americas (n=34) | 29 | 15 | 53 | 3 | 0 | 100 |
| Asia (n=36) | 14 | 6 | 67 | 0 | 14 | 100 |
| Europe (n=41) | 10 | 0 | 85 | 0 | 5 | 100 |
| Oceania (n=13) | 23 | 8 | 69 | 0 | 0 | 100 |
| **Global (n=174)** | **17** | **6** | **70** | **3** | **5** | **100** |
| **World Bank income group** | | | | | | |
| Low income (n=25) | 8 | 4 | 80 | 8 | 0 | 100 |
| Lower middle income (n=45) | 22 | 9 | 62 | 4 | 2 | 100 |
| Upper middle income (n=49) | 27 | 8 | 57 | 2 | 6 | 100 |
| High income (n=54) | 9 | 2 | 81 | 0 | 7 | 100 |
| **Global (n=173)** | **17** | **6** | **69** | **3** | **5** | **100** |

Note:

i) Policy documents were found for 174 of the 194 countries so n=174 for SDG regions but n=173 for World Bank income groups because one country for which policy documents were found (Cook Islands) is not assigned to a World Bank income group.
