# Supplementary material for: Is addressing violence against women prioritised in health policies? Findings from a WHO policies database
Source: PLOS Glob Public Health. 2024 Feb 16;4(2):e0002504. doi: 10.1371/journal.pgph.0002504 (PMC10871498; doi:10.1371/journal.pgph.0002504)
Supplement: S8 Table — (DOCX) [file pgph.0002504.s008.docx]

S8 Table: Proportion of countries that include mental health treatment in policy, by SDG regions and World Bank income groups

|  | **Yes, included**  **(%)** | **Not specified**  **(%)** | **Unclear**  **(%)** | **Unknown - translation not available/usable**  **(%)** | **Total**  **(%)** |
| --- | --- | --- | --- | --- | --- |
| **SDG region** | | | | | |
| Africa (n=50) | 52 | 44 | 2 | 2 | 100 |
| Americas (n=34) | 74 | 26 | 0 | 0 | 100 |
| Asia (n=36) | 56 | 33 | 0 | 11 | 100 |
| Europe (n=41) | 39 | 59 | 0 | 2 | 100 |
| Oceania (n=13) | 31 | 69 | 0 | 0 | 100 |
| **Global (n=174)** | **52** | **44** | **1** | **3** | **100** |
| **World Bank income group** | | | | | |
| Low income (n=25) | 60 | 40 | 0 | 0 | 100 |
| Lower middle income (n=45) | 56 | 40 | 2 | 2 | 100 |
| Upper middle income (n=49) | 59 | 37 | 0 | 4 | 100 |
| High income (n=54) | 41 | 54 | 0 | 6 | 100 |
| **Global (n=173)** | **53** | **43** | **1** | **3** | **100** |

Note:

i) Policy documents were found for 174 of the 194 countries so n=174 for SDG regions but n=173 for World Bank income groups because one country for which policy documents were found (Cook Islands) is not assigned to a World Bank income group.
